# Inferring the functional effect of gene expression changes in signaling pathways

**DOI:** 10.1093/nar/gkt451

**Published:** 2013-06-08

**Authors:** Patricia Sebastián-León, José Carbonell, Francisco Salavert, Rubén Sanchez, Ignacio Medina, Joaquín Dopazo

**Affiliations:** ^1^Department of Computational Genomics, Centro de Investigación Príncipe Felipe (CIPF), Valencia 46012, Spain, ^2^CIBER de Enfermedades Raras (CIBERER), Valencia 46012, Spain, ^3^Genometra S.L., Valencia, Spain and ^4^Functional Genomics Node (INB) at CIPF, Valencia 46012, Spain

## Abstract

Signaling pathways constitute a valuable source of information that allows interpreting the way in which alterations in gene activities affect to particular cell functionalities. There are web tools available that allow viewing and editing pathways, as well as representing experimental data on them. However, few methods aimed to identify the signaling circuits, within a pathway, associated to the biological problem studied exist and none of them provide a convenient graphical web interface. We present PATHiWAYS, a web-based signaling pathway visualization system that infers changes in signaling that affect cell functionality from the measurements of gene expression values in typical expression microarray case–control experiments. A simple probabilistic model of the pathway is used to estimate the probabilities for signal transmission from any receptor to any final effector molecule (taking into account the pathway topology) using for this the individual probabilities of gene product presence/absence inferred from gene expression values. Significant changes in these probabilities allow linking different cell functionalities triggered by the pathway to the biological problem studied. PATHiWAYS is available at: http://pathiways.babelomics.org/.

## INTRODUCTION

Signaling pathways represent the way in which the combined effect of gene activity elicits cell-level responses by activating/deactivating specific functionalities in response to particular stimulus through a chain of intermediate molecules. Different repositories, such as KEGG (1), Reactome (2) and BioCarta (http://www.biocarta.com), contain detailed information about pathways. These repositories offer diagrams that constitute a static representation of the different relationships between the molecules involved in the pathways. Despite its usefulness, this information cannot be edited or easily used for modeling, simulation or any other type of analysis. Therefore, the full exploitation of such information requires of complementary developments that convert such information into a useful tool.

Different software tools have been developed to help visualizing, editing and superimposing gene expression values over the pathway structures (3–9). Such tools build models of pathways typically based on graphical views that represent genes, proteins or metabolites as nodes with edges connecting them that represent different types of functional interactions. However, they are purely descriptive, or, in the best case, they produce a global statistic similar to an enrichment analysis (10). Only a few tools have been recently published addressing the problem of interpreting changes in gene expression within the context of a pathway (11,12). However, these tools have been implemented in the statistical programming language R (http://www.R-project.org), which drastically limits its use only to experienced data analyzers.

Here, we present a new web server, PATHiWAYS, which can be used to easily infer changes in signaling with consequences for cell functionality from the measurements of gene expression values in typical expression microarray case–control experiments. Contrarily to other tools, PATHiWAYS use a probabilistic model to transform differences in gene expression levels into differences in the probability of signal transmission from the nodes that receive the stimulus to the final nodes that trigger the response. In this way, gene expression data, of often difficult interpretation, are transformed into meaningful functional information regarding changes in the different pathway responses triggered by particular stimulus.

## MATERIALS AND METHODS

### The browser framework

The initial web interface appears once the run PATHiWAYS button is pressed. It contains six sections for uploading the experimental data and controlling some aspects of the analysis to be performed. The ‘Species’ section allows choosing between human and mouse species and between three standard Affymetrix platforms in the case of human data and two platforms in the case of mouse data. The ‘Select your data’ section allows uploading the gene expression data to analyze in the context of the pathways. Users can upload normalized matrices of gene expression files (standard output format of many normalization programs that include a first column with probe identifiers and the subsequent columns with their expression values in a number of experiments) as well as raw data as CEL files. The ‘Experimental design’ section allows users to upload another text file containing the labels (case/controls) that define for the individual microarrays. Files in these formats are generated by many software packages, including the Babelomics (http://www.babelomics.org) server (13). An example of colorectal cancer data (14) is pre-loaded and can be used to explore the program functionality. The section ‘Other parameters’ allows selecting the way you want PATHiWAYS summarize the activation probabilities of all the genes mapping belonging to a node (see the description of the algorithm later in the text). The section ‘Pathways’ allows selecting the pathways to be analyzed of a total of 27 pathways that include signal transduction pathways (ERBB, WNT, NOTCH, JAK-STAT, calcium, VEGF, hedgehog signaling and mTOR signaling), signaling molecules and interaction pathways (neuroactive ligand–receptor interaction, cytokine–cytokine receptor, ECM–receptor interaction and cell adhesion molecules), cell growth and death (apoptosis and p53 signaling pathway), cell communication (gap junction and tight junction), endocrine system (insulin, adipocytokine, GNRH and melanogenesis) and immune system (toll-like, B-cell receptor signaling pathway, T-cell receptor signaling pathway, Fc EPSILON RI, antigen processing and chemokine signaling). Finally, the ‘Job’ section allows assigning the Job name, optionally adding a description for the analysis and selecting where to store the job.

### Transforming gene expression data into differences in the probabilities of receptor–effector signal transmission

A simple probabilistic model of the pathway is used to calculate the probabilities for signal transmission from any receptor protein to any final effector protein (taking into account all the intermediate activator and/or repressor proteins in between) using for this the individual probabilities of protein presence/absence. Gene expression values are taken as proxies of gene activity and, consequently, the presence/absence of the corresponding protein (15). More than 10 000 Affymetrix microarrays, obtained from the GEO database (16), have been used to derive the empirical distributions of presence/absence for each probe, which are used to calculate the probability of presence/absence for the probes corresponding to the genes involved in the studied pathways (15). To reduce the number of false positives caused by erroneous probe measurements, we use the 90th percentile of the distribution of the probe activation probabilities as the value of probability of having the gene active as default (although other summarizations are available in the program). When nodes are composed by more than one protein we distinguish two situations: alternative proteins or protein complexes. In the first case, the probability of having the node active is taken as the highest probability of any of the proteins in the node, given that they are supposed to be redundant. In the case of protein complex, all the proteins should be present to guarantee the integrity of the complex. Therefore, the probability of having the node active is conditioned to the lowest probability of all the proteins belonging to the complex. This simplification has proven to be useful in practical terms (17).

Having the individual probabilities of the nodes already estimated, the probability of signal transmission along a stimulus–response circuit can be inferred from the probabilities of activation of all the connecting nodes, taking into account that inhibitor nodes allow signal transmission when they are deactivated. Therefore, a simple product of probabilities (using the principle of inclusion/exclusion when bi- or multi-furcating stretches are present) provides an easy and reasonably accurate approach to estimate the probability of signal transmission sought. However, such probabilities of signal transmission by themselves are meaningless. What is interesting is the comparison of such probabilities in two different conditions (typically cases versus controls). We apply a conventional multi-level analysis (18) that allows detecting which stimulus–response circuits significantly change their probabilities of signal transmission.

### Output

**Figure 1.**
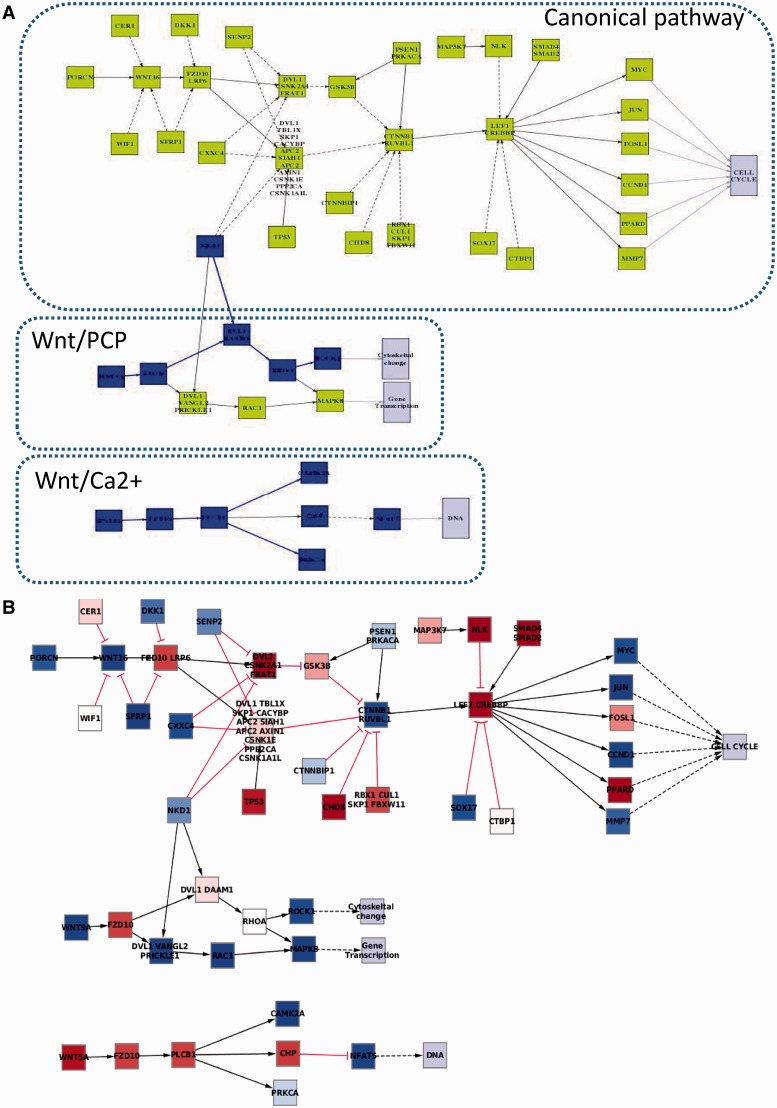
Graphical representation of Wnt pathway as described in the KEGG repository (1). This pathway has a canonical Wnt/β-catenin cascade and two non-canonical pathways, named Wnt/Planar cell polarity (Wnt/PCP) pathway and Wnt/Ca2+ pathway, which conform the complete Wnt signaling pathway (**A**). Here, we represent the Wnt pathway in a gene expression microarray experiment in which colorectal cancer patients are compared with control subjects (14). In the diagrams, nodes represent proteins and edges represent the type of interactions among them. (A) The results provided by PATHiWAYS in which the stimulus–response circuits with significantly decreased activity in cancer patients with respect to healthy control subjects are highlighted in blue. Solid edges indicate activations and dotted edges indicate inhibitions. (**B**) Representation, using the cytoscape program, of the individual gene expression activations (in red) or deactivations (in blue) when comparing the cancer samples with respect to the normal control samples. Edges ended in an arrow indicate activations and edges ended in ‘T’ indicate inhibitions. Despite many genes in many circuits increase or decrease their activity (B), only a few circuits are really affected by these changes (A).

The output consists of a summary of the pathways selected for the analysis with scores of global activity and the number of individual stimulus/response circuits with a significantly altered activity between the cases and the controls. Additionally, for all the pathways, a detailed list of the changes in the activities of all the stimulus–response circuits in the case–control comparison is provided, along with a graphical representation of the multi-level test. Also a graphical representation of the pathway with the stimulus–response circuits color coded according to their corresponding activity changes (calculated as the combined effect of individual gene activities). Figure 1A shows an example with the stimulus–response circuits with significantly decreased activity in the WNT pathway of colorectal cancer patients when compared with control subjects (14). Although many genes in many circuits increase or decrease their activity (Figure 1B), it is clear from the picture that the combined effect of changes in gene expression levels only results on a significant change in the probability of signal transmission only in the Wnt/PCP and Wnt/Ca2+ subpathways (Figure 1A in blue) in the cancer samples with respect to the normal control samples, but they do not affect to the canonical pathway. Actually, this prediction fits with previous observations, which report that Wnt/Ca2+ is downregulated for different cancers, including colorectal cancer (19).

## DISCUSSION

Pathways constitute probably the best representation of biological knowledge of specific cellular processes experimentally validated by hundreds of researchers. This knowledge cumulated over years has been codified into a graphical representation, which is available through different repositories (1,2). In particular, signaling pathways define a system of relationships among the involved gene products in which the interpretation of the final effect caused by different individual changes in gene expression is far from being obvious. For example, it is surprising to realize how often many individual gene expression changes have no effect on stimulus/response circuit activity and vice-versa, as exemplified in Figure 1. Figure 1A summarizes the consequences of the gene activations and deactivations represented in Figure 1B. All the interplay of gene activations and deactivations in the upper part of the pathway has ultimately no effect in the probabilities of signal transmission.

Available web-based graphic tools essentially represent data onto the pathway structure, leaving the functional interpretation to the researcher (3–8). However, the practical applicability of such tools is limited given that signaling is a complex interplay between gene activity and pathway topology with final consequences of difficult evaluation. On the other hand, tools that offer a more sophisticated analysis by means of any type of modeling are stand-alone applications written in the statistical language R of difficult use for non-expert users and provide limited graphical representations (11,12).

PATHiWAYS is a unique web tool that provides an interpretation of the functional consequences of signaling activity changes in a pathway using for this raw gene expression values. PATHiWAYS offers for the first time a rigorous testing framework in a friendly and easy-to-use graphical web-based environment.

The unprecedented increase in the genomic data is not running in parallel with the development of tools for the exploitation of the information contained in such data. This proposal points to an interesting direction: turning data (gene expression measurements) into valuable biological information (actions triggered by signaling pathways). Signaling pathways are on the basis of many diseases and account for many key biological processes. However, the information coded in them remains largely unexploited. Actually there is a lack of tools that use the knowledge of the circuitry that allows signal transduction to provide sophisticated genomic data analysis within a graphical environment. PATHiWAYS offers a straightforward manner of converting genome-scale gene expression data into significant changes in cell signaling (that is, into cell functionality) that can, therefore, be easily related to the specific biological problem studied.

## FUNDING

Funding for open access charge: Spanish Ministry of Economy and Competitiveness (MINECO) [BIO2011-27069 and INNPACTO (IPT-010000-2010-43) CITRUSGENN]; Conselleria de Educacio of the Valencia Community [PROMETEO/2010/001]; National Institute of Bioinformatics (www.inab.org); CIBER de Enfermedades Raras (CIBERER), both initiatives of the ISCIII, MINECO and the Bull Chair in Computational Genomics (http://bioinfo.cipf.es/chair_compgenom).

*Conflict of interest statement.* None declared.
